# The Hypoglycemic Effect of JinQi Jiangtang Tablets Is Partially Dependent on the Palmatine-Induced Activation of the Fibroblast Growth Factor Receptor 1 Signaling Pathway

**DOI:** 10.3389/fphar.2022.895724

**Published:** 2022-07-22

**Authors:** Siming Li, Xiaoling Li, HeMeng Wang, Xinhang Jia, Haoyang Mao, Fangxin Dong, Tingting Zhao, Yuan Gao, Chen Zhang, Ruisong Bai, Ruihao Liu, Lijun Yan, Yubin Ji, Na Zhang, Wenfei Wang

**Affiliations:** ^1^ School of Pharmacy, Harbin University of Commerce, Harbin, China; ^2^ College of Life Sciences, Tarim University, Alar, China; ^3^ College of Life Sciences, Northeast Agricultural University, Harbin, China; ^4^ Aier School of Ophthalmology, Central South University, Changsha, China; ^5^ College of Food Engineering, Harbin University of Commerce, Harbin, China

**Keywords:** JinQi Jiangtang tablets (JQJTT), hypoglycemic effect, fibroblast growth factor receptor (FGFR1), palmatine (PAL), SPR fishing

## Abstract

JinQi Jiangtang tablet (JQJTT) is a Chinese patent medicine that has been shown to be beneficial for patients with diabetes both preclinically and clinically; however, the molecular mechanism underlying the effects of JQJTT remains unclear. In this study, surface plasmon resonance fishing was employed to identify JQJTT constituent molecules that can specifically bind to fibroblast growth factor receptor 1 (FGFR1), leading to the retrieval of palmatine (PAL), a key active ingredient of JQJTT. *In vivo* and *in vitro* experiments demonstrated that PAL can significantly stimulate FGFR1 phosphorylation and upregulate glucose transporter type 1 (GLUT-1) expression, thereby facilitating glucose uptake in insulin resistance (IR) HepG2 cells as well as alleviating hyperglycemia in diabetic mice. Our results revealed that PAL functions as an FGFR1 activator and that the hypoglycemic effect of JQJTT is partially dependent on the PAL-induced activation of the FGFR1 pathway. In addition, this study contributed to the understanding the pharmacodynamic basis and mechanism of action of JQJTT and provided a novel concept for future research on PAL.

## 1 Introduction

JinQi Jiangtang tablet (JQJTT) is a compound formula composed of three traditional herbal medicines, namely, *Coptis chinensis* (Ranunculaceae), *Astragalus membranaceus* (Leguminosae), and *Lonicera japonica* (Caprifoliaceae). It is an improved formula based on the classic “Qianjin Huanglian pill” recorded in the Bei Ji Qian Jin Yao Fang during the Tang Dynasty in China and is the first Chinese patent medicine approved by the China Food and Drug Administration (Han, 2009; Liu et al., 2019) for the treatment of type 2 diabetes. JQJTT contains a variety of active ingredients, including alkaloids, polysaccharides, saponins, and flavonoids, that allow it to comprehensively regulate the body’s metabolic environment by improving glucose and lipid metabolism and reducing inflammation (Cao et al., 2010; Wang et al., 2017). Nevertheless, the mechanisms underlying the effects of JQJTT remain unclear.

Fibroblast growth factor 21 (FGF21) exerts its biological functions predominantly *via* fibroblast growth factor receptor 1 (FGFR1) (Gong, 2014; Kilkenny and Rocheleau, 2016). Over recent years, research attention has increasingly focused on the indispensable role of the FGF21/FGFR1 signaling pathway in regulating glucose and lipid metabolism (Marseglia et al., 2019; Ye et al., 2019). In the present study, we found that the phosphorylation level of FGFR1 and that of its intracellular substrate FRS2, as well as the protein levels of glucose transporter type 1 (GLUT-1), were significantly increased in the livers of mice with streptozotocin (STZ)-induced diabetes after gavage administration of JQJTT. These results suggested that, like FGF21, there may be molecules in JQJTT that can regulate glucose and lipid metabolism by activating FGFR1. Thus, using FGFR1 as the target, we sought to identify JQJTT constituent molecules that could bind to FGFR1 using surface plasmon resonance (SPR)-based molecular fishing (Cao et al., 2016; Chen et al., 2018). Analysis of the recovered solution led to the identification of palmatine (PAL), one of the main components of JQJTT, as an FGFR1-targeting compound with a high affinity for the receptor. PAL was found to regulate glucose absorption in insulin resistance (IR) HepG2 cells and whole-body glucose homeostasis in mice. Consistent with the hypoglycemic effect of FGF21, PAL could also stimulate FGFR1/FRS2 phosphorylation both *in vivo* and *in vitro*, as well as activate the AKT and AMPK pathways, downstream effectors of FGF21/FGFR1/FRS2 signaling.

Combined, our results demonstrated that PAL is an FGFR1 activator and that the hypoglycemic effect of JQJTT is partially dependent on PAL-induced activation of the FGFR1 pathway. This study contributed to the understanding of the pharmacodynamic basis and mechanism of action of JQJTT and provided a novel perspective for future research on PAL.

## 2 Materials and Methods

### 2.1 Reagents and Antibodies

JQJTT (190210) was provided by Longshunrong Pharmaceutical Factory of Tianjin Zhongxin Pharmaceutical Group Co., Ltd (Tianjin, China). PAL (CAS: 3486-67-7, purity >98%) was provided by Sichuan Vikqi Biotechnology Co., Ltd (Sichuan, China). FGF21 protein was obtained from Northeast Agricultural University. STZ and insulin were purchased from Sigma-Aldrich Co., Ltd (St. Louis, MO, United States). PD173074 was purchased from Selleck Chemicals (Houston, TX, United States). The glucose detection kit was obtained from Shanghai Yuanye Bio-Technology Co., Ltd (Shanghai, China). Trizol and the cDNA reverse transcription kit were purchased from Thermo Fisher Scientific (Carlsbad, CA, United States). SYBR Green qPCR Mix was provided by Shandong Sikejie Biotechnology Co., Ltd (Shandong, China). Primary antibodies targeting phosphorylated (p)-FGFR1 (ab173305), FRS2 (ab137458), AMPK (ab207442), p-AMPK (ab133448), and β-actin (ab8227) were purchased from Abcam (Cambridge, United Kingdom); those targeting p-FRS2 (YP0805), p-AKT (YT0185), and AKT (YP0006) were obtained from ImmunoWay (California,United States); and that targeting FGFR1 (9740T) was purchased from CST (Boston, MA, United States). Secondary antibodies were obtained from Absin Biotechnology Co., Ltd (Shanghai, China).

### 2.2 Animals and Treatment

KM mice (6 weeks old, 20 ± 5 g) were obtained from Changchun Yisi Experimental Animal Technology Co., Ltd (Changchun, China). The mice were kept in a controlled environment (temperature: 24 ± 2°C; relative humidity: 50%–60%; 12-h light/12-h dark cycle) and had free access to food and water for 1 week. Type 2 diabetes models were established by STZ injection (50 mg/kg) twice in 3 days and were fed with high-fat diet at the same time. Fasting blood glucose level greater than 16.7 mmol/L was regarded as successful modeling. Model mice were then randomly divided into four groups (*n* = 12 per group), three of which received intragastric administration of JQJTT of differing concentrations (JQ-H: 800 mg/kg JQJTT; JQ-M: 400 mg/kg JQJTT; and JQ-L: 200 mg/kg JQJTT) for four consecutive weeks, while one (the model group) was fed the same amount of normal saline. The normal control group was also fed the same amount of normal saline. Changes in blood glucose in the mice were measured weekly. To further test the effect of PAL, mice were randomly divided into six groups (*n* = 12), PAL-H (80 mg/kg), PAL-M (40 mg/kg), PAL-L (20 mg/kg), and FGF21 (0.5 mg/kg) groups. Animals were treated via intraperitoneal injection for four consecutive weeks, when the control groups received an intraperitoneal injection of an equal amount of normal saline. Subsequently, the fasting blood glucose changes in mice were tested weekly. On day 28, the intraperitoneal glucose tolerance test (IPGTT) was performed. Furthermore, the mice were euthanized, and liver tissues were harvested for western blotting and quantitative PCR (qPCR) analysis. All experiments were carried out in strict accordance with the recommendations of the Guide for the Care and Use of Laboratory Animals of the National Institutes of Health and were approved by the Ethics Review Committee of Harbin University of Commerce (No. HSDYXY-2020006).

### 2.3 Blood Glucose Assay

During drug treatment, once weekly, fasting blood glucose was directly measured in the tail vein of mice after a 12-h fast using a glucometer (Glucotrend; Roche Diagnostics Ltd, Mannheim, Germany). On day 28, changes in blood glucose in the mice were detected 1, 2, 4, and 8 h after PAL treatment. For the IPGTT, mice were fasted overnight (12–16 h) and then intraperitoneally injected with 20% glucose (2 g/kg). At 0, 30, 60, and 120 min after injection, blood was collected from the tail vein for the determination of blood glucose concentrations. Assessment was based on the area under the glucose curve (AUC), which was calculated using the following equation: AUC = 0.5 × (BG0 + BG30)/2 + 0.5 × (BG30 + BG60)/2 + 1× (BG60 + BG120)/2. The glucose uptake rate was calculated as follows: glucose concentration (mmol/L) = OD of the sample/OD of the standard × 5 mmol/L; glucose consumption rate (%) = [(C blank group glucose-C administration group glucose)/C blank group glucose] × 100%.

### 2.4 Cell Culture and Treatments

HepG2 cells were a kind donation from the Heilongjiang University of Traditional Chinese Medicine (Heilongjiang, China). The cells were cultured in Dulbecco’s modified Eagle’s medium (DMEM) (Gibco, Carlsbad, California, United States) supplemented with 10% fetal bovine serum (FBS) (Gibco, Carlsbad, California, United States) and 1% penicillin–streptomycin (Thermo Fisher Scientific) at 37°C and with 5% CO_2_.

#### 2.4.1 Generation of a HepG2 Cell Model of Insulin Resistance (IR HepG2 Cells)

HepG2 cells in the logarithmic growth phase were seeded in a 96-well plate at a density of 5×10^4^ cells/ml, allowed to adhere, washed twice with DMEM without FBS, and then incubated for 24 h in 200 μl of DMEM containing a final concentration of 1 × 10^–6^ mol/L insulin to induce IR.

#### 2.4.2 Drug Treatment

IR HepG2 cells were treated or not with FGF21 or PAL previously diluted with DMEM without FBS. Treatments included an FGF21 group (0.5 mg/kg) and three PAL groups (PAL-H [80 μg/ml], PAL-M [40 μg/ml], and PAL-L [20 μg/ml]). The blank group and the model group were treated with the same concentrations of dimethyl sulfoxide (DMSO) (Solarbio, Shanghai, China). Treatments lasted for 24 h.

#### 2.4.3 Glucose Consumption Test

Then, 24 h after administration, 2 μl of the culture medium supernatant was placed in a glucose working solution, and the glucose content was detected using the glucose oxidase–peroxidase method.

### 2.5 Isolation and Identification of Fibroblast Growth Factor Receptor 1–Binding Molecules in JinQi Jiangtang Tablet

Fishing experiments were performed on a BIACORE T200 instrument at 25°C. FGFR1 protein was immobilized on all four channels of a CM5 chip following a standard EDC/NHS protocol. JQ-R tablets (JQ-R is a mixture of refined extracts from *Coptis chinensis*, *Astragalus membranaceus*, and *Lonicera japonica*, the three main constituents of JQJTT) were fully dissolved in water, and the insoluble residue was pelleted by centrifugation and discarded. Then, the supernatant was then injected at a flow rate of 10 μl/min. All four flow cells were used for analyte capture and recovery. The bound material was eluted with 0.5% trifluoroacetic acid.

For the kinetics experiment, six or seven analytes were serially diluted twofold, prepared in the running buffer, and injected at a flow rate of 30 μl/min onto the CM5 chips, followed by 1 min of dissociation data acquisition. To obtain more extensive off-rate decay data, an additional injection of the analyte with the third-highest concentration was included in each experiment. All covalent surfaces were regenerated with 10 mM glycine–HCl (pH 2.5, GE HealthcaMolecular Dockingre). All experiments were conducted at 25°C.

### 2.6 Molecular Docking

The 3D structures of the FGFR1 (PDB ID: 4V05) and FGF21 (PDB ID: 5VAQ) proteins were downloaded from the RCSB PDB database (https://www.rcsb.org/), and the 2D structure of PAL was obtained from the PubChem database (https://pubch em.ncbi.nlm.nih.gov). Open Babel 2.4.1 software was used to convert PAL 2D structure results to “mol2” format. PyMOL 2.4.0 software was used for receptor dehydrogenation and ligand separation. The data were then imported into AutoDockTools 1.5.6 for pretreatment, including hydrogenation, to identify the active pocket in the docking region of the receptor. Using AutoDock Vina 1.1.2 software, FGFR1 was molecularly docked with PAL and the C-terminal domain of FGF21 (https://www.rcsb.org/structure/5VAQ). The docking results were analyzed using PyMOL software.

### 2.7 Quantitative Reverse Transcription PCR

Total RNA was extracted from cells and animal livers using Trizol reagent and then reverse transcribed into cDNA using Oligo-dT primers. qPCR was used to measure the relative mRNA expression levels of GLUT-1 in cells or livers. Relative mRNA expression levels were calculated using the 2^−ΔΔCT^ method with *GAPDH* serving as the reference gene. The sequences of the primers used for qPCR were as follows: GAPDH forward, 5′-CCT​TCC​GTG​TTC​CTA​CCC​C-3′ and GAPDH reverse, 5′-GCC​CAG​GAT​GCC​CTT​TAG​TG-3′; GLUT-1 forward, 5′-CAT​CAA​TGC​CCC​CCA​GAA-3′ and GLUT-1 reverse, 5′-AAG​CGG​CCC​AGG​ATC​AG-3′.

### 2.8 Western Blotting

Total protein was extracted from cells and liver tissue using radioimmunoprecipitation assay lysis buffer. Protein concentrations were measured using a UV spectrophotometer. Equal amounts of protein were separated by sodium dodecyl sulfate–polyacrylamide gel electrophoresis, transferred to a polyvinylidene fluoride membrane (Millipore, Massachusetts, Germany), blocked with 5% skimmed milk for 1 h, and then incubated first with antibodies targeting FGFR1, p-FGFR1, FRS2, p-FRS2, AKT, p-AKT, AMPK, p-AMPK, and β-actin overnight at 4°C and then with horseradish peroxidase-labeled secondary antibody for 1 h at room temperature. Protein bands were detected using an enhanced chemiluminescence reagent (Cytiva, Logan, Utah, United States) in the dark.

### 2.9 Statistical Analysis

Statistical analysis was performed using GraphPad Prism 8.0 (GraphPad Software, Inc., San Diego, CA, United States). Data are presented as means ± SD. The *t*-test was used to analyze differences between two groups. Comparisons among three or more groups were performed using one-way ANOVA followed by Tukey’s *post hoc* test. A *p*-value < 0.05 was considered significant.

## 3 Results

### 3.1 JinQi Jiangtang Tablet Reduces Blood Glucose and Stimulates Fibroblast Growth Factor Receptor 1 Phosphorylation in Mice With Streptozotocin-Induced Diabetes

**FIGURE 1:**
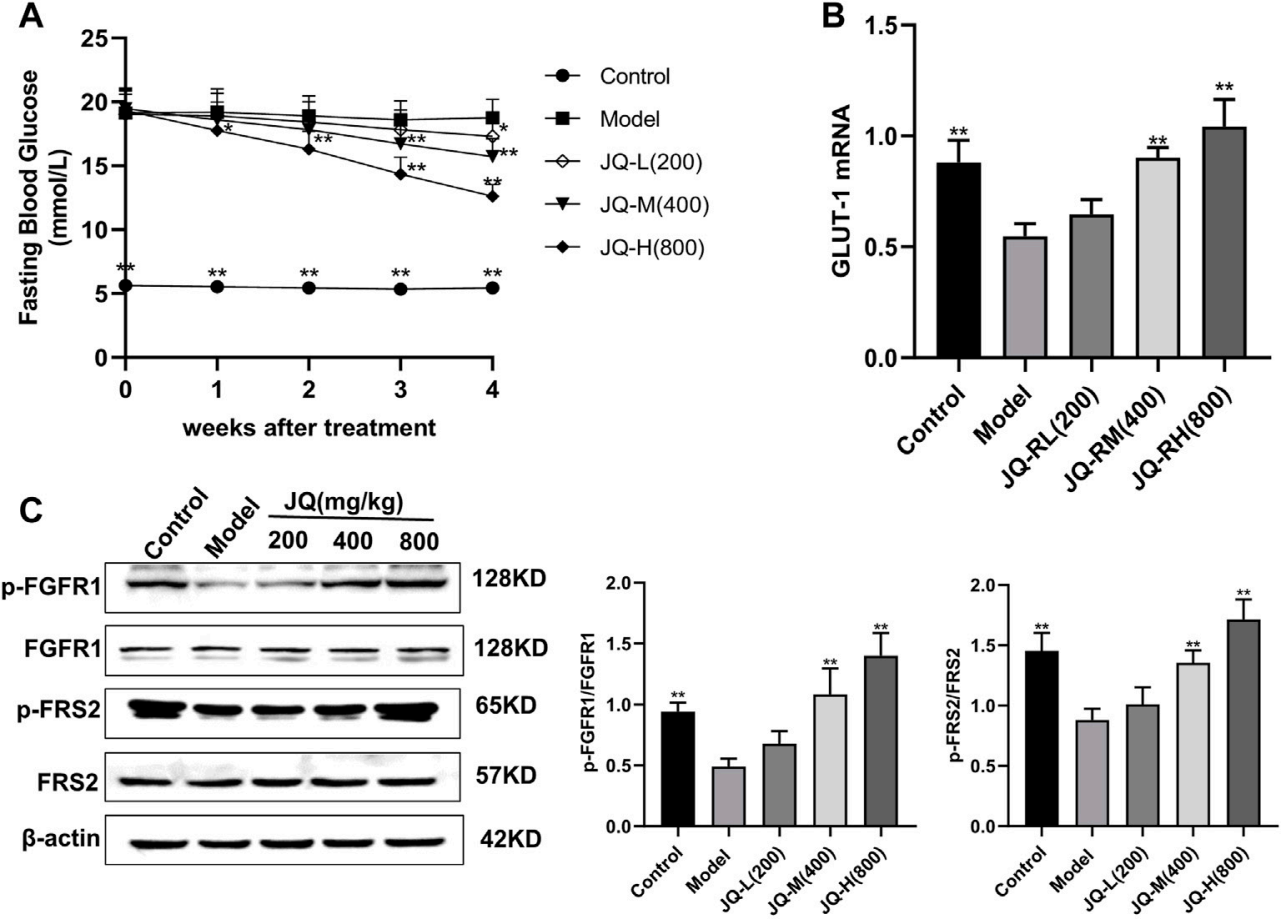
The effect of JinQi Jiangtang tablet (JQJTT) on the fibroblast growth factor receptor 1 (FGFR1) pathway in mice with streptozotocin (STZ)-induced diabetes. **(A)** The fasting blood glucose level in each group. **(B)** The relative mRNA level of glucose transporter type 1 (GLUT-1) in the livers of mice in each group. **(C)** The protein expression levels of p-FGFR1 and FGFR1 (p-FGFR1/total FGFR1) and those of p-FRS2 and FRS2 (and p-FRS2/total FRS2) as quantified using western blot. Data are presented as means ± SD, *n* = 12. *p < 0.05, **p < 0.01, *versus* the model group.

In mice with STZ-induced diabetes, fasting blood glucose levels were significantly decreased after 4 weeks of treatment with JQ-R (Figure 1A), whereas the phosphorylation level of FGFR1 and that of its intracellular substrate FRS2, as well as the protein levels of GLUT-1, were markedly increased. Moreover, these effects were JQ-R dose-dependent (Figures 1B,C). These results suggested that the FGFR1 pathway may exert hypoglycemic effects and that there may be effective molecules in JQJTT that directly activate FGFR1.

### 3.2 Identification and Isolation of the Fibroblast Growth Factor Receptor 1–Targeting Compound Palmatine in JinQi Jiangtang Tablet

**FIGURE 2:**
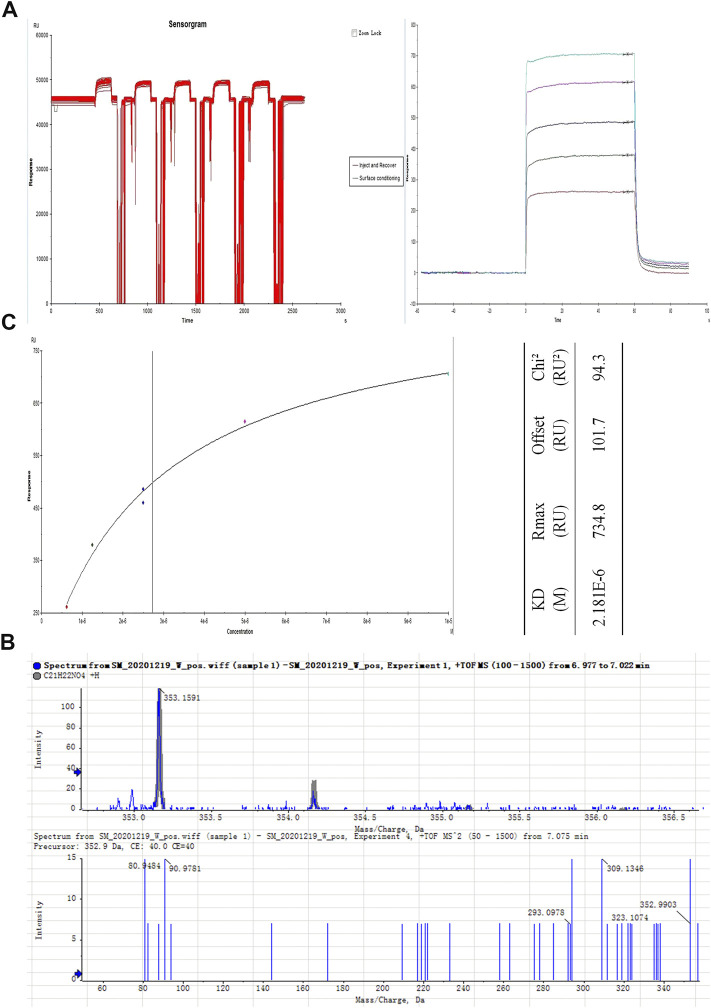
Isolation and identification of the JinQi Jiangtang tablet (JQJTT)-derived fibroblast growth factor receptor 1 (FGFR1) activator palmatine (PAL). **(A)** The variation in response values for PAL/FGFR1 during the fishing experiment. **(B)** Identification of PAL by mass spectrometry. **(C)** PAL/FGFR1 affinity characterization.

To explore the mechanism *via* which JQJTT activates the FGFR1 pathway, an SPR assay was used to identify active ingredients in JQJTT that can directly target FGFR1. Figure 2A illustrates the variation in response values during the fishing experiment. The recovery solution was subjected to UPLC–MS/MS, and the resultant chromatogram is shown in Figure 2B. HR-ESI-MS data in positive ion mode showed a peak at *m*/*z* 353.1549 [M + Na]^+^ (calcd. 352.1549), indicating that the molecular formula of the detected ingredient was C21H22NO4, which identified it as PAL. To verify that the candidate compound indeed had a strong affinity for FGFR1, an affinity assay was performed using Biacore T200 Evaluation Software Version 3.0. The resulting sensogram and fitting curve indicated that the KD value for the interaction between PAL and FGFR1 was 2.181 × 10^–6^ kcal/mol, thus representing a good affinity between the two molecules (Figure 2C).

### 3.3 Palmatine Within JinQi Jiangtang Tablet Molecularly Interacts With Fibroblast Growth Factor Receptor 1

**FIGURE 3:**
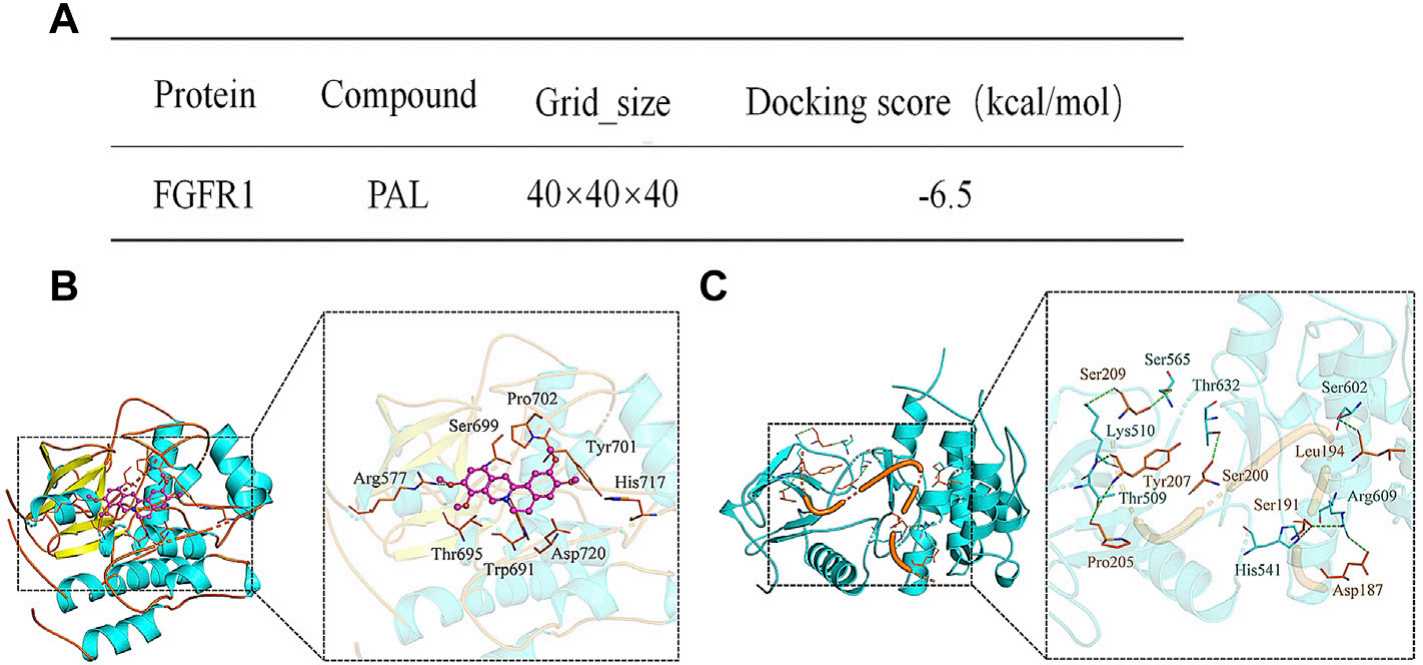
Analysis of the molecular interaction between palmatine (PAL) and fibroblast growth factor receptor 1 (FGFR1). **(A)** The binding energy between FGFR1 and PAL. **(B)** A three-dimensional representation of the docking between FGFR1 and PAL. **(C)** A three-dimensional representation of the docking between FGFR1 and FGF21.

The interaction energy and binding sites between PAL and FGFR1 were predicted using Molecular Docking analysis. The predicted score for interaction energy (−6.5 kcal/mol) indicated that PAL could act as an activator of FGF21 and therefore also the FRFR1 signaling pathway (Figure 3A). Furthermore, as shown in Figure 3B, the amino acid residue Arg577 of FGFR1 tended to form hydrogen bonds in its interaction with PAL, while the amino acid residues Ser699, Pro702, Tyr701, His717, Asp720, Trp691, and Thr695 in FGFR1 enabled hydrophobic interactions with PAL. Figure 3C shows the binding pattern between FGFR1 and its ligand FGF21. Amino acid residues Ser565, Thr632, Ser602, Arg609, His541, Thr509, and Lys510 in FGFR1 interacted with Ser209, Tyr207, Pro205, Ser200, Ser191, Leu194, and Asp187 in FGF21 through hydrogen bonds. As a whole, these data showed that the site for FGFR1/PAL binding differs from that for the FGFR1/FGF21 combination.

### 3.4 Palmatine Promoted Glucose Absorption by Activating the Fibroblast Growth Factor Receptor 1 Pathway in IR HepG2 Cells

**FIGURE 4:**
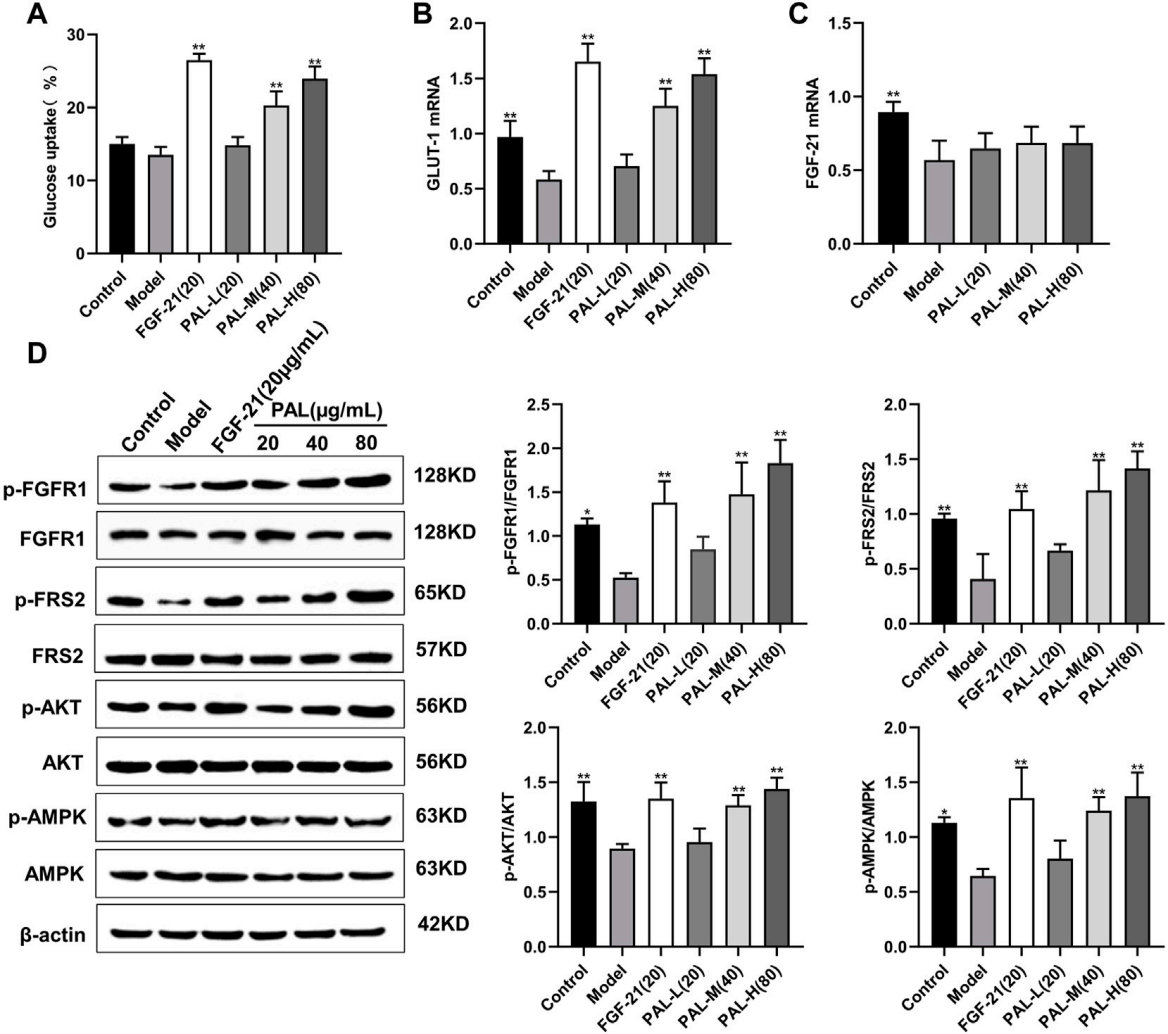
The effect of palmatine (PAL) on the fibroblast growth factor receptor 1 (FGFR1) pathway in IR HepG2 cells. **(A)** glucose uptake by IR HepG2 cells after PAL treatment. **(B)** the relative mRNA level of glucose transporter type 1 (GLUT-1) in IR HepG2 cells of each group. **(C)** the relative mRNA level of FGF21 in IR HepG2 cells of each group. **(D)** quantitative analysis of the expression levels of p-FGFR1 and FGFR1 (p-FGFR1/total FGFR1), p-FRS2 and FRS2 (p-FRS2/total FRS2), p-AKT and AKT (p-AKT/total AKT), and p-AMPK and AMPK (p-AMPK/total AMPK) using western blot. Data are presented as means ± SD, *n* = 9. *p < 0.05, **p < 0.01, *versus* the model group.

To detect the effects of PAL *in vitro*, we examined the effect of PAL on the FGFR1 pathway in IR HepG2 cells. As expected, we found that PAL could activate FGFR1 and its downstream substrate FRS2 in a dose-dependent manner *via* improving IR HepG2 cell glucose absorption and GLUT-1 mRNA expression (Figures 4A,B) without affecting the FGF21 mRNA level (Figure 4C). This effect was not seen in IR model cells. In addition, PAL also promoted the activation of the AKT and AMPK pathways, which are the main pathways that regulate glucose and lipid metabolism downstream of FGFR1 (Figure 4D). These findings demonstrated that PAL has hypoglycemic properties.

### 3.5 Treatment With a Fibroblast Growth Factor Receptor 1 Inhibitor Blocked the Effects of Palmatine in IR HepG Cells

**FIGURE 5:**
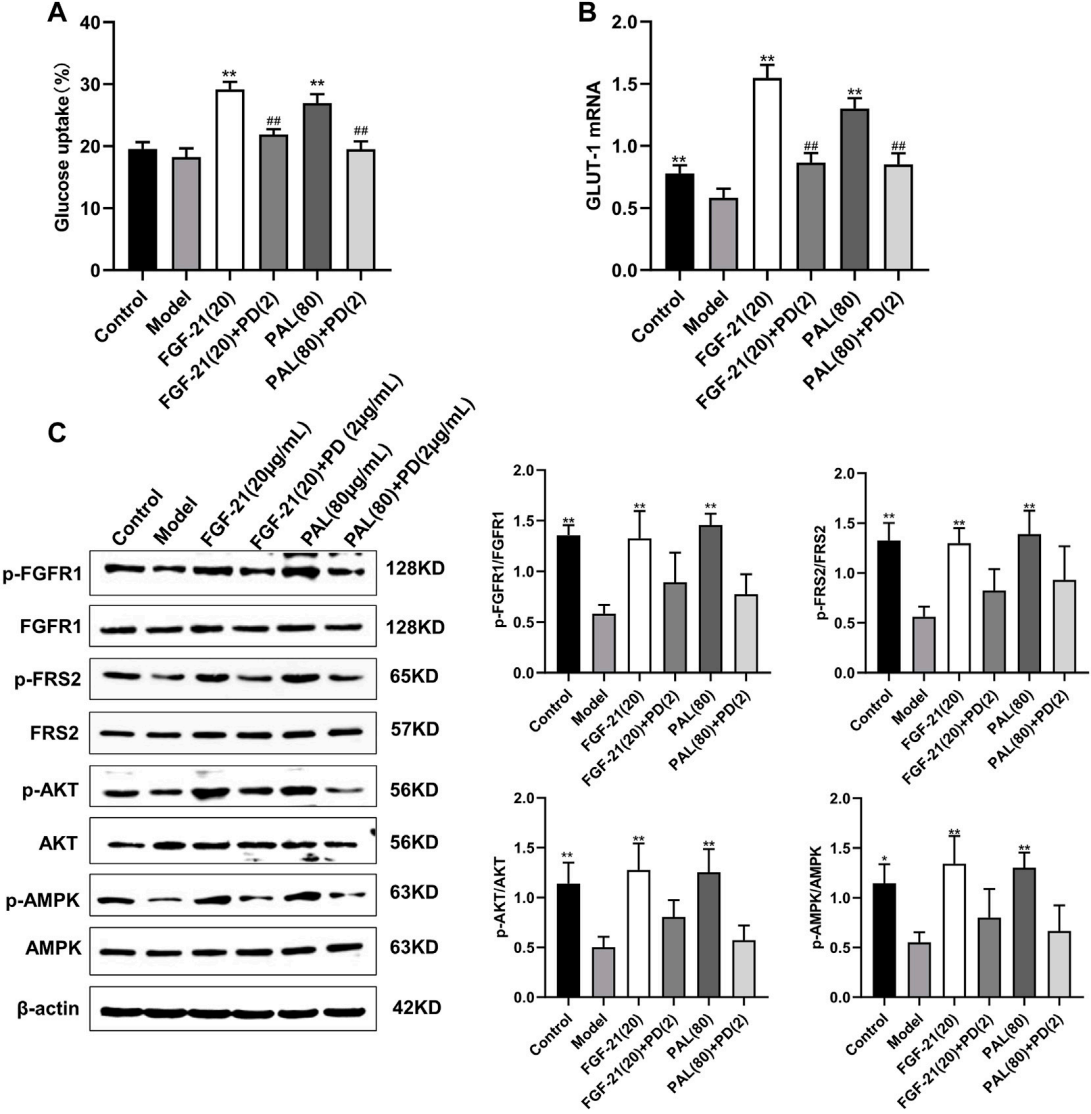
Treatment with an fibroblast growth factor receptor 1 (FGFR1) inhibitor blocked the effects of palmatine (PAL) in IR HepG2 cells. **(A)** glucose uptake by IR HepG2 cells after FGFR1 inhibitor treatment. **(B)** the relative mRNA level of glucose transporter type 1 (GLUT-1) in IR HepG2 cells treated with the FGFR1 inhibitor. **(C)** quantitative analysis of the expression levels of p-FGFR1 and FGFR1 (p-FGFR1/total FGFR1), p-FRS2 and FRS2 (p-FRS2/total FRS2), p-AKT and AKT (p-AKT/total AKT), and p-AMPK and AMPK (p-AMPK/total AMPK) by western blot. Data are presented as means ± SD, *n* = 9. *p < 0.05, **p < 0.01, *versus* the model group; ^##^ *p* < 0.01, *versus* the untreated group.

In IR HepG2 cells, treatment with the FGFR1 inhibitor PD1730749 significantly inhibited the PAL-mediated promotion of glucose absorption (Figure 5A) and GLUT-1 mRNA expression (Figure 5B). Likewise, the PAL-mediated activation of FGFR1 and its downstream AKT and AMPK signaling pathways were also suppressed after FGFR1 inhibitor treatment (Figure 5C).

### 3.6 Palmatine Ameliorated Hyperglycemia by Activating the Fibroblast Growth Factor Receptor 1 Signaling Pathway in Mice With Streptozotocin-Induced Diabetes

**FIGURE 6:**
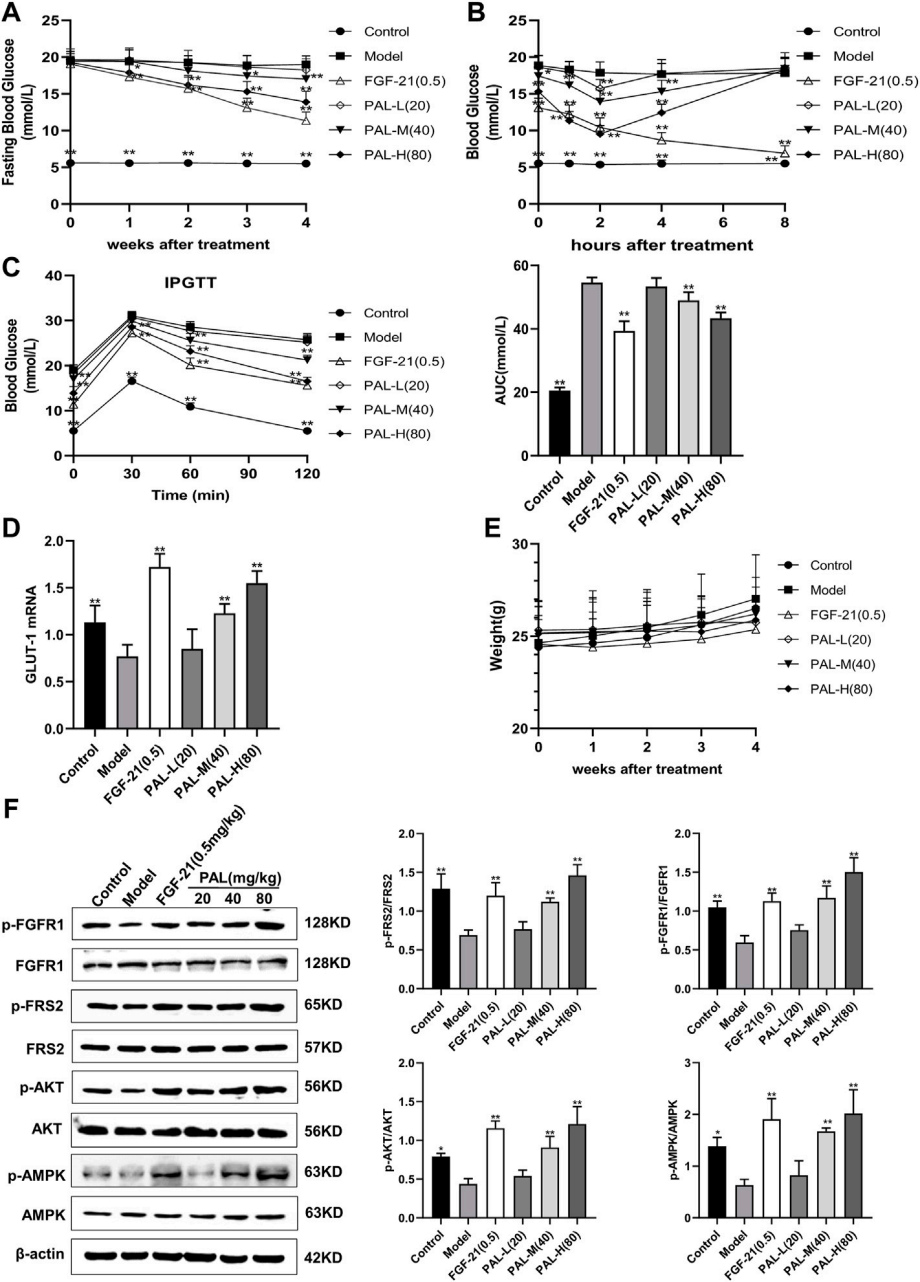
The effect of palmatine (PAL) on the fibroblast growth factor receptor 1 (FGFR1) pathway in mice with streptozotocin (STZ)-induced diabetes. **(A)** fasting blood glucose in mice treated with PAL for 4 weeks. **(B)** changes in blood glucose levels over time in mice on day 28 of PAL treatment. **(C)** intraperitoneal glucose tolerance test (IPGTT) after 4 weeks of PAL treatment. **(D)** the relative mRNA level of glucose transporter type 1 (GLUT-1) in the livers of mice in each group. **(E)** weight changes in mice treated with PAL for 4 weeks **(F)** quantitative analysis of the expression levels of p-FGFR1 and FGFR1 (p-FGFR1/total FGFR1), p-FRS2 and FRS2 (p-FRS2/total FRS2), p-AKT and AKT (p-AKT/total AKT), and p-AMPK and AMPK (p-AMPK/total AMPK) using western blot. Data are presented as means ± SD, *n* = 12. *p < 0.05, **p < 0.01, *versus* the model group.

As shown in Figure 6A, after 4 weeks of PAL administration, fasting blood glucose levels in mice with STZ-induced diabetes were significantly decreased in each PAL dosage group. On day 28 of administration, blood glucose was measured 1, 2, 4, and 8 h after PAL administration. The results revealed that, in STZ-treated mice, the hypoglycemic effect of each PAL dose was greatest after 2 h of treatment (Figure 6B). On the final day of administration, the IPGTT test indicated that after 2 h of PAL intervention, blood glucose levels and the AUC in each PAL dose group underwent a pronounced reduction (Figure 6C). At the same time, the GLUT-1 mRNA level in the liver showed a marked increase after PAL treatment; however, no significant change was observed in the level of FGF21 mRNA (Figures 6D,E). In addition, western blot results indicated that PAL improved blood glucose levels in STZ-treated mice while simultaneously activating FGFR1 and its downstream AMPK and AKT signaling pathways (Figure 6F), suggesting that PAL also plays a hypoglycemic role *in vivo via* binding to FGFR1 and activating its downstream signaling.

## 4 Discussion

Like in many Chinese herbal medicines, numerous chemical components have been identified in JQJTT using HPLC–ESI–Q-TOF/MS, including iridoid glycosides, phenolic acids, alkaloids, flavonoids, and saponins (Sun et al., 2014; Chang et al., 2015). This abundance of constituents makes it extremely difficult to identify the effective components and pharmacological mechanism of JQJTT, which hinders the application and further development of this drug.

Studies have shown that JQJTT can regulate the endocrine environment *via* activating multiple signaling pathways (Cao et al., 2019; Liu et al., 2019). In addition, one study reported that JQJTT can improve glucose and lipid metabolism and alleviate IR by promoting AMPK pathway activity (Gao et al., 2014), while a different study found that JQJTT can stimulate glucose absorption mainly *via* the PI3K/AKT signaling and alleviate inflammation by inhibiting the NF-κB pathway (Liu et al., 2017). However, the substances involved in these effects of JQJTT remain unclear, as do the underlying mechanisms.

FGFR1, a member of the fibroblast growth factor receptor family, is the key receptor mediating the biological functions of FGF21 (Gong, 2014; Kilkenny and Rocheleau, 2016). Over recent years, increasing research attention has focused on FGF21 owing to its potential as a therapeutic agent for the treatment of metabolic diseases (Domouzoglou et al., 2015; Jimenez et al., 2018). Several studies have reported that FGF21 can bind to the extracellular domain of FGFR1 and stimulate its phosphorylation and consequent activation. Once activated, FGFR1 exerts positive effects on hypoglycemia and lipid reduction by activating the AKT, AMPK, and NF-κB signaling pathways (Yu et al., 2016; Salminen et al., 2017; Guo et al., 2018; Wang et al., 2018).

The similarity between JQJTT and FGF21 in clinical effects and pharmacological mechanisms suggests that JQJTT may contain molecules that can mimic FGF21 function and activate FGFR1. To assess this possibility, we first evaluated the effects of JQJTT on FGFR1 and its downstream substrate FRS2 in the liver of mice with STZ-induced diabetes. We found that, like FGF21, JQJTT could significantly promote FGFR1 activity and FRS2 phosphorylation in the liver of model mice (Figure 1), implying that a molecule or molecules in JQJTT can bind to FGFR1 and mimic the biological role of FGF21 in modulating glucose and lipid metabolism. To further identify the relevant molecule(s), an SPR-based fishing method was used to isolate molecules with high affinity for FGFR1, resulting in the isolation and identification of PAL as a FGFR1 activator. In addition, SPR assay results indicated that the KD value for the interaction between PAL and FGFR1 was 2.181 × 10^–6^ kcal/mol, indicative of a good affinity between the two molecules.

PAL, an isoquinoline alkaloid rich in rhizoma cupids, is one of the main components that can be detected in serum after oral JQJTT administration (Jin et al., 2018; Liu et al., 2019; Shang et al., 2020). PAL is mainly used in the treatment of surgical infection and inflammatory diseases (Long et al., 2019). Recent studies have also shown that PAL can exert ameliorative effects in metabolic diseases; however, the mechanisms underlying these effects of PAL have yet to be elucidated (Ekeuku et al., 2020; Tian et al., 2020). To determine whether PAL could modulate glucose or lipid metabolism by activating FGFR1, we examined the effects of PAL on FGFR1 and its downstream signaling pathways both *in vitro* and *in vivo.* The results showed that PAL could greatly stimulate FGFR1 and FRS2 phosphorylation and promote GLUT-1 expression and glucose absorption in IR hepG2 cells in a dose-dependent manner (Figure 3). In addition, PAL administration activated the AKT and AMPK pathways, which are the main pathways that regulate glucose and lipid metabolism downstream of FGFR1 (Figure 3C), while these biological effects were significantly inhibited following treatment with the FGFR1 inhibitor PD173074 (Figure 4). Similar results were obtained *in vivo*, which provides further evidence that PAL exerts hypoglycemic effects *via* directly activating FGFR1.

In particular, we found that the binding sites involved in FGFR1/PAL interaction differed from those required for the FGFR1/FGF21 combination (Figures 3B,C). The FGF family comprises a total of 23 members. However, there are only seven members in the FGFR family. Thus, each receptor may bind a different ligand and transmit different signals (Zhang et al., 2006; Dai et al., 2019). Moreover, the different signal transduction pathways associated with the different receptors can result in diverse biological effects (Li, 2019). For instance, FGF21, FGF1, and FGF19 share the same FGFR, but the respective biological functions and associated mechanisms are not identical. We speculate that the reason why the hypoglycemic activity of PAL was not identical to that of FGF21 is that the ligand-binding sites involved in interactions with the receptor led to different changes in the intracellular structure of FGFR1, which would be expected to result in the activation of different downstream signaling pathways (Suh et al., 2014; Chen et al., 2021; Talukdar and Kharitonenkov, 2021; Sancar et al., 2022). In addition, the FGFR1 signaling pathway encompasses a variety of biological functions, including the regulation of blood lipid levels, the relief of inflammation, and antioxidative effects. Thus, the physiological processes mediated by PAL *via* FGFR1 merit further investigation.

In conclusion, we found that PAL could bind to FGFR1 and directly activate its downstream signaling pathway. Our findings contribute to a deeper understanding of the pharmacodynamic basis and mechanism of action of JQJTT and provide a foundation for further research on the pharmacological effects of PAL.

## Data Availability

The datasets presented in this study can be found in online repositories. The names of the repository/repositories and accession number(s) can be found in the article/supplementary material.

## References

[B1] Cao H. Ren M. Guo L. Shang H. Zhang J. Song Y. (2010). JinQi-Jiangtang Tablet, a Chinese Patent Medicine, for Pre-diabetes: a Randomized Controlled Trial. Trials 11, 27. 10.1186/1745-6215-11-27 20214831 PMC2842259

[B2] Cao Y. Li Y. H. Lv D. Y. Chen X. F. Chen L. D. Zhu Z. Y. (2016). Identification of a Ligand for Tumor Necrosis Factor Receptor from Chinese Herbs by Combination of Surface Plasmon Resonance Biosensor and UPLC-MS. Anal. Bioanal. Chem. 408 (19), 5359–5367. 10.1007/s00216-016-9633-6 27225174

[B3] Cao Y. Yao G. Sheng Y. Yang L. Wang Z. Yang Z. (2019). JinQi Jiangtang Tablet Regulates Gut Microbiota and Improve Insulin Sensitivity in Type 2 Diabetes Mice. J. Diabetes Res. 2019, 1872134. 10.1155/2019/1872134 30733971 PMC6348821

[B4] Chang Y. X. Ge A. H. Donnapee S. Li J. Bai Y. Liu J. (2015). The Multi-Targets Integrated Fingerprinting for Screening Anti-diabetic Compounds from a Chinese Medicine Jinqi Jiangtang Tablet. J. Ethnopharmacol. 164, 210–222. 10.1016/j.jep.2015.02.018 25698248

[B5] Chen H. Li J. Zhang D. Zhou X. Xie J. (2021). Role of the Fibroblast Growth Factor 19 in the Skeletal System. Life Sci. 265, 118804. 10.1016/j.lfs.2020.118804 33245964

[B6] Chen L. Lv D. Chen X. Liu M. Wang D. Liu Y. (2018). Biosensor-based Active Ingredients Recognition System for Screening STAT3 Ligands from Medical Herbs. Anal. Chem. 90 (15), 8936–8945. 10.1021/acs.analchem.8b01103 29953204

[B7] Dai S. Zhou Z. Chen Z. Xu G. Chen Y. (2019). Fibroblast Growth Factor Receptors (FGFRs): Structures and Small Molecule Inhibitors. Cells 8 (6), 614. 10.3390/cells8060614 PMC6627960 31216761

[B8] Domouzoglou E. M. Naka K. K. Vlahos A. P. Papafaklis M. I. Michalis L. K. Tsatsoulis A. (2015). Fibroblast Growth Factors in Cardiovascular Disease: The Emerging Role of FGF21. Am. J. Physiol. Heart Circ. Physiol. 309 (6), H1029–H1038. 10.1152/ajpheart.00527.2015 26232236 PMC4747916

[B9] Ekeuku S. O. Pang K. L. Chin K. Y. (2020). Palmatine as an Agent Against Metabolic Syndrome and Its Related Complications: A Review. Drug. Des. Devel. Ther. 14, 4963–4974. 10.2147/DDDT.S280520 PMC7680161 33235437

[B10] Gao L. H. Liu Q. Liu S. N. Chen Z. Y. Li C. N. Lei L. (2014). A Refined-JinQi-JiangTang Tablet Ameliorates Prediabetes by Reducing Insulin Resistance and Improving Beta Cell Function in Mice. J. Ethnopharmacol. 151 (1), 675–685. 10.1016/j.jep.2013.11.024 24286962

[B11] Gong S. G. (2014). Isoforms of Receptors of Fibroblast Growth Factors. J. Cell. Physiol. 229 (12), 1887–1895. 10.1002/jcp.24649 24733629

[B12] Guo D. Xiao L. Hu H. Liu M. Yang L. Lin X. (2018). FGF21 Protects Human Umbilical Vein Endothelial Cells against High Glucose-Induced Apoptosis via PI3K/Akt/Fox3a Signaling Pathway. J. Diabetes Complicat. 32 (8), 729–736. 10.1016/j.jdiacomp.2018.05.012 29907326

[B13] Han C. (2009). Comparison of Anti-hyperglycemic Effect of Inorganic Constituents and Organic in Traditional Chinese Medicine, Jinqi Compound Recipe. Biol. Trace Elem. Res. 131 (1), 55–61. 10.1007/s12011-009-8344-7 19247586

[B14] Jimenez V. Jambrina C. Casana E. Sacristan V. Muñoz S. Darriba S. (2018). FGF21 Gene Therapy as Treatment for Obesity and Insulin Resistance. EMBO Mol. Med. 10 (8), e8791. 10.15252/emmm.201708791 29987000 PMC6079533

[B15] Jin H. Mou J. J. Xia N. Ding F. F. Chen F. Deng Y. R. (2018). Identification and Analysis of Absorbed Components in Rat Plasma after Oral Administration of Jinqi Jiangtang Tablets by UPLC-ESI-MS. Drug Eval. Res. 41 (12), 2227–2231. 10.7501/j.issn.1674-6376.2018.12.017

[B16] Kilkenny D. M. Rocheleau J. V. (2016). The FGF21 Receptor Signaling Complex: Klothoβ, FGFR1c, and Other Regulatory Interactions. Vitam. Horm. 101, 17–58. 10.1016/bs.vh.2016.02.008 27125737

[B17] Li X. (2019). The FGF Metabolic axis. Front. Med. 13 (5), 511–530. 10.1007/s11684-019-0711-y 31495905 PMC7102389

[B18] Liu Q. Liu S. Gao L. Sun S. Huan Y. Li C. (2017). Anti-diabetic Effects and Mechanisms of Action of a Chinese Herbal Medicine Preparation JQ-R *In Vitro* and in Diabetic KKAy Mice. Acta. Pharm. Sin. B 7 (4), 461–469. 10.1016/j.apsb.2017.04.010 28752031 PMC5518656

[B19] Liu Y. Wang A. Wen L. Yang Z. Yang X. Zhang X. (2019). A Chinese Medicine Formula (Jinqi Jiangtang Tablet): A Review on its Chemical Constituents, Quality Control, Pharmacokinetics Studies, Pharmacological Properties and Clinical Applications. J. Ethnopharmacol. 236, 1–8. 10.1016/j.jep.2019.02.038 30802612

[B20] Long J. Song J. Zhong L. Liao Y. Liu L. Li X. (2019). Palmatine: a Review of its Pharmacology, Toxicity and Pharmacokinetics. Biochimie 162, 176–184. 10.1016/j.biochi.2019.04.008 31051209

[B21] Marseglia G. Lodola A. Mor M. Castelli R. (2019). Fibroblast Growth Factor Receptor Inhibitors: Patent Review (2015-2019). Expert Opin. Ther. Pat. 29 (12), 965–977. 10.1080/13543776.2019.1688300 31679402

[B22] Salminen A. Kauppinen A. Kaarniranta K. (2017). FGF21 Activates AMPK Signaling: Impact on Metabolic Regulation and the Aging Process. J. Mol. Med. Berl. 95 (2), 123–131. 10.1007/s00109-016-1477-1 27678528

[B23] Sancar G. liu S. Gasser E. Alvarez J. G. Moutos C. Kim K. (2022). FGF1 and Insulin Control Lipolysis by Convergent Pathways. Cell. Metab. 34 (1), 171–e6. 10.1016/j.cmet.2021.12.004 34986332 PMC8863067

[B24] Shang X. F. Yang C. J. Morris-Natschke S. L. Li J. C. Yin X. D. Liu Y. Q. (2020). Biologically Active Isoquinoline Alkaloids Covering 2014-2018. Med. Res. Rev. 40 (6), 2212–2289. 10.1002/med.21703 32729169 PMC7554109

[B25] Suh J. M. Jonker J. W. Ahmadian M. Goetz R. Lackey D. Osborn O. (2014). Endocrinization of FGF1 Produces a Neomorphic and Potent Insulin Sensitizer. Nature 513 (7518), 436–439. 10.1038/nature13540 25043058 PMC4184286

[B26] Sun S. Xie Z. S. Liu E. H. Yan Y. T. Xu X. J. Li P. (2014). Chemical Profiling of Jinqi Jiangtang Tablets by HPLC-ESI-Q-TOF/MS. Chin. J. Nat. Med. 12 (3), 229–240. 10.1016/S1875-5364(14)60039-X 24702812

[B27] Talukdar S. Kharitonenkov A. (2021). FGF19 and FGF21: In NASH We Trust. Mol. Metab. 46, 101152. 10.1016/j.molmet.2020.101152 33383173 PMC8085573

[B28] Tian X. Zhang Y. Li H. Li Y. Wang N. Zhang W. (2020). Palmatine Ameliorates High Fat Diet Induced Impaired Glucose Tolerance. Biol. Res. 53 (1), 39. 10.1186/s40659-020-00308-0 32928312 PMC7491132

[B29] Wang H. Guo L. Shang H. Ren M. Wang X. Wang D. (2017). JinqiJiangtang Tablets for Pre-diabetes: A Randomized, Double-Blind and Placebo-Controlled Clinical Trial. Sci. Rep. 7 (1), 11190. 10.1038/s41598-017-11583-5 28894283 PMC5593818

[B30] Wang N. Li J. Y. Li S. Guo X. C. Wu T. Wang W. F. (2018). Fibroblast Growth Factor 21 Regulates Foam Cells Formation and Inflammatory Response in Ox-LDL-Induced THP-1 Macrophages. Biomed. Pharmacother. 108, 1825–1834. 10.1016/j.biopha.2018.09.143 30372888

[B31] Ye L. Wang X. Cai C. Zeng S. Bai J. Guo K. (2019). FGF21 Promotes Functional Recovery after Hypoxic-Ischemic Brain Injury in Neonatal Rats by Activating the PI3K/Akt Signaling Pathway via FGFR1/β-Klotho. Exp. Neurol. 317, 34–50. 10.1016/j.expneurol.2019.02.013 30802446

[B32] Yu Y. He J. Li S. Song L. Guo X. Yao W. (2016). Fibroblast Growth Factor 21 (FGF21) Inhibits Macrophage-Mediated Inflammation by Activating Nrf2 and Suppressing the NF-Κb Signaling Pathway. Int. Immunopharmacol. 38, 144–152. 10.1016/j.intimp.2016.05.026 27276443

[B33] Zhang X. Ibrahimi O. A. Olsen S. K. Umemori H. Mohammadi M. Ornitz D. M. (2006). Receptor specificity of the fibroblast growth factor family. The complete mammalian FGF family. J. Biol. Chem. 281 (23), 15694–15700. 10.1074/jbc.M601252200 16597617 PMC2080618

